# Secondary sex ratio in relation to exposures to polychlorinated biphenyls, dichlorodiphenyl dichloroethylene and methylmercury

**DOI:** 10.1080/22423982.2017.1406234

**Published:** 2017-12-01

**Authors:** Clara Amalie Gade Timmermann, Anna L. Choi, Maria Skaalum Petersen, Flemming Nielsen, Esben Budtz-Jørgensen, Pál Weihe, Philippe Grandjean

**Affiliations:** ^a^ Department of Environmental Medicine, University of Southern Denmark, Odense C, Denmark; ^b^ Department of Environmental Health, Harvard T.H. Chan School of Public Health, Boston, MA, USA; ^c^ Department of Health Research and Policy, Stanford University, Stanford, CA, USA; ^d^ Department of Occupational Medicine and Public Health, The Faroese Hospital System, Tórshavn, Faroe Islands; ^e^ Faculty of Health Sciences, Center of Health Science, University of The Faroe Islands, Tórshavn, Faroe Islands; ^f^ Section of Biostatistics, University of Copenhagen, Copenhagen K, Denmark

**Keywords:** Contaminants, DDE, Faroe islands, PCB, methylmercury, pilot whale, sex ratio

## Abstract

This study was undertaken to assess the potential impact of maternal exposures to polychlorinated biphenyl (PCB), dichlorodiphenyl dichloroethylene (DDE) and methylmercury on the secondary sex ratios (the ratio of male to female live births) over a span of 23 years. The study includes prospective data from three Faroese birth cohorts, with a total of 2,152 healthy mother–child dyads recruited between 1986 and 2009. The Faroe Islands is a subarctic fishing community, where pilot whale meat and blubber are part of the traditional marine diet. Exposures were measured in maternal hair, serum or umbilical cord blood. Confounder adjusted logistic regression models were used to assess the associations between maternal exposures and the secondary sex ratio. A doubling in ΣPCB, *p,p’*-DDE and mercury concentrations were associated with increased odds by 8% (95% CI = 0–16%), 7% (95% CI = 0–14%) and 9% (95% CI = 2–17%), respectively, of giving birth to a boy. In conclusion, maternal exposure to ΣPCB, DDE and methylmercury was associated with a slightly *increased* secondary sex ratio. The impact of paternal exposures could not be taken into account and deserves attention.

## Introduction

The number of male live births exceeds the number of female live births [1], but in parts of the world the ratio between the two has decreased over the past decades [2]. Endocrine disruptive effects from persistent organic pollutants (POPs) on hormonal systems during conception and pregnancy have been suggested as explanations for sex ratio changes [3].

Following the Seveso accident in Italy in 1976, where a wide area was contaminated with dioxin, the secondary sex ratio (the male/female ratio at birth) was found to decline among the exposed population [4]. This discovery led to an increased focus on the potential of organochlorines to alter the sex ratio and maternal exposure to other organochlorines, i.e. polychlorinated biphenyls (PCBs), was found to be associated with a decreased male/female birth ratio [5,6]. However, other studies found no such association [7–9], nor has an association been found between a similar organochlorine substance, dichlorodiphenyl dichloroethylene (DDE) and sex ratio [6,9]. Furthermore, changes in sex ratios observed over time are apparently unrelated to increased environmental contamination in the Arctic [10].

PCBs and DDE are lipophilic, highly persistent and prone to bioaccumulate in adipose tissues and in the food chain [11,12]. Due to concerns over their toxicity and persistence in the environment, these chemicals have been banned since the 1970s in industrialised countries. However, dichlorodiphenyl trichloroethane (DDT), which breaks down to DDE in the environment, is still ubiquitously used, especially in Africa [11]. Also, human exposure endures because of residues in products manufactured before the ban and the environmental persistence and bioaccumulative characteristics of these compounds. Due to their lipophilic properties, PCBs and DDE are found in fatty fish, which is a major source of exposure to humans [13].

In addition to possible effects of POPs, a recent study found that severe environmental methylmercury contamination in Minamata, Japan was associated with a decreased secondary sex ratio [14]. Methylmercury is formed from inorganic mercury by aquatic microbes and is neurotoxic [15]. Similar to PCB and DDE, marine diet is an important source of methylmercury exposure [16].

In this study, we aim to examine whether prenatal exposures to methylmercury and the major POPs, PCB and DDE are associated with the secondary sex ratio in a population with a tradition of high consumption of marine food.

## Materials and methods

### Study area and cohort recruitment

The Faroe Islands is a marine community located in the North Atlantic, where the traditional diet holds a high content of marine food that includes whale meat and blubber, thereby exposing the Faroese to high concentrations of environmental chemicals [17–19]. In recent decades, the serum-concentrations of environmental chemicals have decreased in pregnant women, perhaps, in part, due to public advisories about high-risk foods [19]. Thus, due to differences in dietary habits and changes over time, a wide span of exposures can be covered in the Faroese population.

Over three decades, three Faroese birth cohorts were formed as part of the Children’s Health and Environment in the Faroe Islands (CHEF) studies. The first cohort (Cohort 1) was formed between 1986 and 1987 [16,20], one (Cohort 3) was formed between 1997 and 2000 [21–23] and the most recent cohort, with necessary exposure data (Cohort 5), was formed between 2007 and 2009 [24–26].

In Cohort 1, a blood sample for PCB and *p,p’*-DDE analyses was obtained from the umbilical cord, while a maternal hair sample for mercury analysis was obtained immediately after birth. For Cohort 3, hair and blood samples were taken from the mother between weeks 34 and 36 of pregnancy, while, for Cohort 5, hair and blood samples were taken from the mother 2 weeks post-term. Information about child sex, maternal age at birth, parity (0, 1, 2, 3+) and pre-pregnancy body mass index (BMI: weight/height^2^) was retrieved from subject charts and hospital records.

Written informed consent was obtained from all women included in the study. The CHEF studies were performed in accordance with the Helsinki declaration and were approved by the Faroese ethical review committee and the institutional review board at Harvard T.H. Chan School of Public Health.

### Exposure assessment

All PCB and DDE analyses were conducted at the University of Southern Denmark. Serum concentrations of major PCB congeners and *p,p’*-DDE in Cohorts 3 and 5 were conducted by gas chromatography with electron capture detection as previously described [21]. PCB concentrations were expressed in relation to the total lipid concentration determined using the Cypress Diagnostics kit (Langdorp, Belgium) [21]. The limit of detection (LOD) for PCB congeners and *p,p’*-DDE in these analyses was 0.03 ng/mL, which corresponds to 0.003 μg/g lipid with an average lipid concentration of 10 g/L [27].

PCBs and *p,p’*-DDE analyses in Cohort 1 cord blood were performed by the same method, as modified to allow for analysis of whole blood [15]. Valid quantification of lipid concentrations in the cord whole blood from Cohort 1 was not possible, but lipid concentrations in cord blood are low and less variable than those in non-fasting adult serum and, as before, an average lipid concentration of 3 g/L was used for converting the volume-based results into lipid based results (μg/g lipid) [15]. The median LOD for *p,p’*-DDE and PCB congeners CB-138, CB-153 and CB-180 in the Cohort 1 analyses was approximately 0.016 μg/g lipid [15]. In samples from all three cohorts, values below the LOD were replaced with LOD/2.

The accuracy of the method was assessed by participation in the German Quality Assessment programme (G-EQUAS) organised by the German Society of Occupational Medicine. Use of quality control samples verified that the results from the three cohorts were comparable.

The three major PCB congeners, CB-138, CB-153 and CB-180, account for approximately 50% of the total PCB concentration [17,28] and a simplified ΣPCB concentration was, therefore, calculated as the sum of congeners CB-138, CB-153 and CB-180, multiplied by 2.

For the mercury analysis, maternal hair samples of at least 100 mg were cut close to the root on the occipital area, tied with cotton string, and saved in small plastic bags [29]. All samples were analysed by standard atomic absorption techniques [16,25,30]. The LOD was 5 ng/g. In this population, the total mercury in hair is almost equal to the methylmercury and the mercury measures were, therefore, used as a proxy for the methylmercury exposure [31].

### Statistical analyses

**Figure 1.**
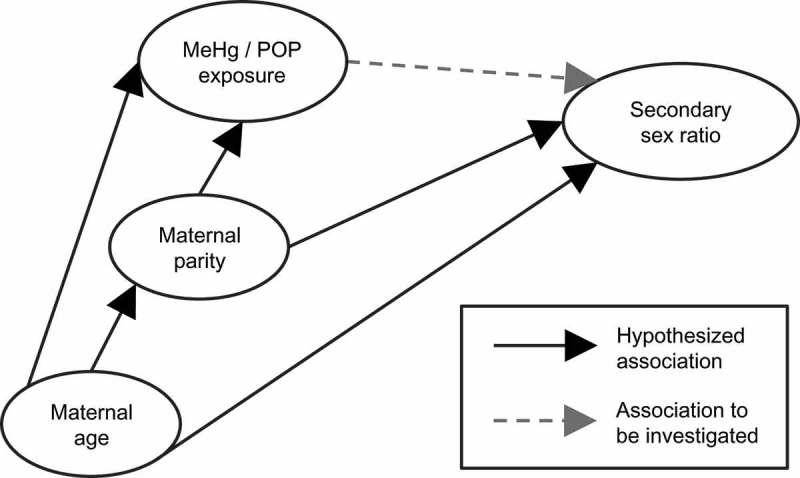
Hypothesised association between methylmercury (MeHg)/POP exposures and secondary sex ratio, including potential confounding paths depicted in a Directed Acyclic Graph (DAG).

Potential confounders were identified using a Directed Acyclic Graph (DAG, Figure 1), based on known associations with sex ratio [32–34], methylmercury [29] and POPs [6,35]. Associations between exposures (ΣPCB and *p,p’*-DDE) and the secondary sex ratio were tested one exposure at a time in marginal and confounder adjusted logistic regression models. The odds are p/(1–p), which is identical to the probability of a boy divided by the probability of a girl. Thus, the odds is equal to the sex ratio. Therefore, results of the logistic regression can be directly interpreted as multiplicative effects on the sex ratio. Distributions of ΣPCB and *p,p’*-DDE concentrations were log_10_-transformed to reduce the influence of outlying values. All regression analyses were performed with cluster-robust standard errors to adjust for dependence between mothers being included with more than one child.

Analyses were performed both with all three cohorts combined and stratified by cohort. When all cohorts were combined, the analyses were adjusted for the effect of cohort to account for cohort differences. An interaction term between cohort and the exposure were tentatively included to test association differences between cohorts. The assumption of a linear association between the log-transformed exposures and the odds ratio (OR) for having a boy was tested tentatively by including non-log-transformed exposure measures.

Since the Cohort 1 exposure measures were only summarily adjusted for lipids, we also performed sensitivity analyses adjusting for maternal pre-pregnancy BMI.

All analyses were performed using Stata version 14.2 (StataCorp, College Station, TX).

## Results

A total of 2,152 mother–child dyads were included in the study; 1,022 from Cohort 1, 640 from Cohort 3 and 490 from Cohort 5. Twin births were excluded, but 39 mothers were included with two singleton births; three mothers were included twice in Cohort 1, nine were included twice in Cohort 3, two were included twice in Cohort 5 and 25 mothers were included once in both Cohort 3 and Cohort 5. Furthermore, 18 girls from Cohort 1 later became mothers to children in Cohort 5 and four boys from Cohort 1 became fathers to children from Cohort 5, one of which also had a mother from Cohort 1.

Characteristics of the women from the three cohorts are shown in Table 1. Among the women included in Cohort 1, slightly more girls than boys were born, whereas more boys than girls were born among the women included in Cohorts 3 and 5. Concentrations of both ΣPCB and *p,p’*-DDE were found to be higher in Cohort 3 compared to Cohorts 1 and 5 (Table 1). However, maternal lipid based serum concentrations have been shown to be 1.7 times those found in the corresponding cord serum samples [36] and the lower concentrations in Cohort 1 can, thus, be attributed to the measures being performed on cord blood rather than maternal serum. Methylmercury concentrations were found to be the highest in Cohort 1 and lowest in Cohort 5. In Cohort 1, *p,p’*-DDE or one of three PCB compounds were below the LOD in 107 samples, but only in five samples were all compounds below the LOD. In Cohorts 3 and 5, only four samples were found with *p,p’*-DDE or one of three PCB compounds below the LOD and none had all compounds below LOD. None of the methylmercury concentrations were below the detection limits. After log-transformation, ΣPCB and *p,p’*-DDE concentrations were strongly correlated (r=0.9) and each of the two POPs were moderately correlated with methylmercury (r=0.4).

**Table 1.** Cohort characteristics.

		Cohort 1		Cohort 3		Cohort 5	
Continuous variables		N	Median (25^th^; 75^th^ percentile)	N	Median (25^th^; 75^th^ percentile)	N	Median (25^th^; 75^th^ percentile)
Cord blood ΣPCB (μg/g lipid)		1,010	0.65 (0.36; 1.07)				
Maternal serum ΣPCB (μg/g lipid)				607	1.18 (0.75; 1.93)	487	0.42 (0.25; 0.77)
Cord blood *p,p’*-DDE (μg/g lipid)		1,010	0.28 (0.14; 0.51)				
Maternal serum *p,p’*-DDE (μg/g lipid)				607	0.54 (0.31; 0.97)	487	0.13 (0.07; 0.29)
Maternal hair-mercury (μg/g)		1,019	4.49 (2.52; 7.66)	609	2.20 (1.22; 3.96)	488	0.71 (0.43; 1.10)
Maternal age (years)		1,022	26.9 (23.2; 30.9)	640	30.1 (25.9; 33.2)	490	30.6 (26.7; 34.5)
Maternal BMI (kg/m^2^)		834	21.6 (20.2; 23.5)	640	23.0 (21.2; 25.6)	489	23.7 (21.2; 26.0)
*Categorical variables*		n/N	%	n/N	%	n/N	%
Child sex	Girls	512/1022	50.1	297/640	46.4	239/490	48.8
	Boys	510/1022	49.9	343/640	53.6	251/490	51.3
Maternal parity	0	354/1021	34.7	176/640	27.5	148/488	30.3
	1	353/1021	34.6	211/640	33.0	170/488	34.8
	2	187/1021	18.3	169/640	26.4	109/488	22.3
	≥ 3	127/1021	12.4	84/640	13.1	61/488	12.5

In marginal logistic regression analyses, ΣPCB, *p,p’*-DDE and methylmercury concentrations were found to be significantly associated with an increased sex ratio. Due to the log-transformed exposures, ORs were calculated for a doubling of the exposures. Thus, a doubling in ΣPCB was associated with a 9% increase, a doubling in *p,p’*-DDE with a 8% increase and a doubling in methylmercury with a 10% increase of the sex ratio for the combined cohorts (Table 2). However, when adjusting the analyses for parity, maternal age and time between childbirth and blood sampling in Cohort 5, the associations were slightly attenuated and the association between ΣPCB and child sex was of borderline significance; a doubling in ΣPCB was associated with an 8% increase of the sex ratio, while it was 7% for a doubling of *p,p’*-DDE and 9% for a doubling of the methylmercury concentration (Table 2). When stratifying the analyses by cohort, ΣPCB and *p,p’*-DDE exposures were significantly associated with increased sex ratio in both marginal and adjusted analyses in Cohort 1. The same tendencies, although somewhat weaker, were seen in Cohort 3. No clear associations were seen in Cohort 5 (Table 2). For methylmercury, the strongest association with sex ratio was seen in Cohort 5 in both the unadjusted and adjusted analyses, while less clear tendencies in the same direction were seen for Cohorts 1 and 3. Still, the differences between cohorts had p_interation_ values of 0.38 for ΣPCB, 0.35 for *p,p’*-DDE and 0.41 for methylmercury.

**Table 2.** Odds ratio for having a boy with each doubling of prenatal exposures to ΣPCB, *p,p’*-DDE and mercury.

		Cohorts combined^a^		Cohort 1		Cohort 3		Cohort 5	
		N	OR (95% CI)	N	OR (95% CI)	N	OR (95% CI)	N	OR (95% CI)
Marginal analyses	ΣPCB	2,104	1.09 (1.01; 1.17)	1,010	1.12 (1.01; 1.24)	607	1.13 (0.98; 1.31)	487	0.99 (0.85; 1.15)
	*p,p’*-DDE	2,104	1.08 (1.01; 1.14)	1,010	1.12 (1.03; 1.22)	607	1.08 (0.95; 1.21)	487	0.99 (0.88; 1.12)
	Mercury	2,116	1.10 (1.02; 1.18)	1,019	1.08 (0.97; 1.20)	609	1.07 (0.95; 1.21)	488	1.21 (1.03; 1.42)
Adjusted analyses^b^	ΣPCB	2,101	1.08 (1.00; 1.16)	1,009	1.14 (1.02; 1.27)	607	1.10 (0.94; 1.29)	485	0.95 (0.81; 1.11)
	*p,p’*-DDE	2,101	1.07 (1.00; 1.14)	1,009	1.13 (1.03; 1.24)	607	1.05 (0.93; 1.20)	485	0.97 (0.86; 1.10)
	Mercury	2,113	1.09 (1.02; 1.17)	1,018	1.08 (0.97; 1.20)	609	1.05 (0.92; 1.18)	486	1.21 (1.02; 1.43)

^a^ Adjusted for cohort.

^b^ Adjusted for maternal parity and maternal age. Analyses including Cohort 5 were additionally adjusted for time between childbirth and blood sampling.

Adjustment for maternal BMI slightly weakened the associations between the POPs and sex, likely as a result of a reduced number of observations included in the analyses.

When including non-log-transformed exposure measures along with the log-transformed exposure measures, the non-log-transformed exposure measures did not show any statistically significant associations, thus indicating that log-linearity is a reasonable assumption.

## Discussion

Contrary to our *a priori* hypothesis, we found that prenatal exposure to ΣPCB, DDE and methylmercury was associated with slightly *increased* odds of a male birth when combining all cohorts and adjusting for potential confounding. However, the strength of the associations varied across the cohorts.

**Figure 2.**
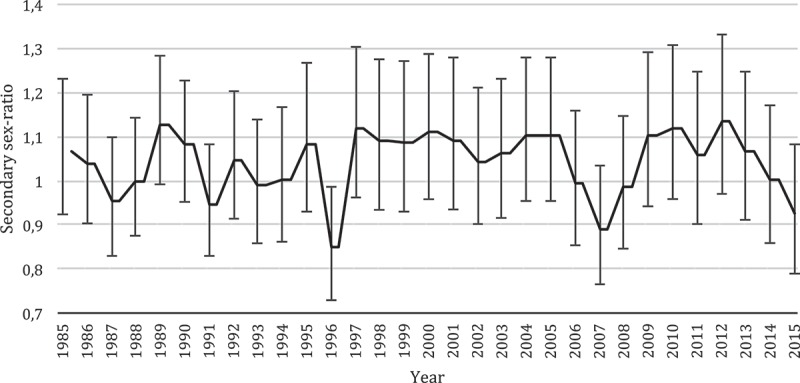
Secondary sex ratio (95% CI) in the Faroe Islands from 1985 to 2015 [35].

Since this study is based on data from three cohorts, exposures were assessed at different times, thus possibly being affected by temporal trends of other origin. However, when examining associations between exposures and sex ratio, we adjusted for cohort in the regression analyses and any temporal change or differences in exposure assessment should, therefore, have no more than a negligible effect. The exposures generally seemed to decrease over time, although part of the decrease in ΣPCB and DDE between Cohorts 3 and 5 may have been due to the post-parturition blood draw in Cohort 5 [35]. However, more likely, the decrease reflects the decreasing popularity of whale blubber consumption. The reduction in exposure levels among the Faroese, especially in pregnant women, is considered a successful outcome of two decades of public health communication regarding marine pollutants [19]. However, no concurrent overall trends in the secondary sex ratio have been observed in the Faroe islands demographic data over the past 20 years [37] (Figure 2).

The median DDE concentrations were much lower among the Faroese women than in a study of pregnant Mexican women in 1998 [38]. The median PCB concentrations in Cohorts 1 and 3 were higher than among American adults in the NHANES 2001–2002 data and among women delivering in two eastern Slovakian areas in 2002–2004, whereas, in Cohort 5, the median PCB concentration was higher than in NHANES, but lower than in one of the two Slovakian areas [39,40]. The median hair mercury concentrations among the Faroese women were higher than in a 1993–1998 cohort of new mothers from Massachusetts, US [41] and the median hair mercury concentrations in Cohorts 1 and 3 were also higher than among a group of Japanese mothers examined in 2002–2003, but in Cohort 5 the median hair mercury concentration was lower than among the Japanese mothers [42].

In accordance with our results, a previous study also found tendencies towards increased secondary sex ratio at higher maternal PCB exposure, although these findings were not statistically significant [43]. Accordingly, a recent review concluded that there is no convincing evidence of an association between ΣPCB and secondary sex ratio [44], although some of the studies included in the review did report a significantly decreased sex ratio at increased PCB exposure [5,6].

In addition to the maternal exposure, paternal exposure to POPs may also affect the secondary sex ratio [7–9], although the direction of the association also differed between studies. Furthermore, a recent study has shown that, among fertile Faroese men, serum concentrations of DDE and PCB were associated with a reduced Y:X sperm ratio [45]. However, the present study encompassed maternal exposure levels only and, therefore, did not take into account any potential effects of paternal exposure. It is possible that the effect of paternal exposure could be opposite to that associated with maternal exposure and that these effects may counteract [3]. Thus, obtaining information about both maternal and paternal exposure would be optimal when examining associations with sex ratio.

The association observed between methylmercury exposure and secondary sex ratio is in contrast to a study of methylmercury contamination in Japan, where methylmercury was associated with a *decreased* secondary sex ratio [14]. However, the Japanese study was ecological, with no direct measures of methylmercury and, thus, the findings could be affected by measurement error and by paternal rather than maternal exposure. Other evidence has not reported any clear association with methylmercury exposure.

As a further complication, it may be that a sensitive window of vulnerability exists with regard to the sex-dimorphic viability of a conceptus affected by environmental toxicants. Before conception, some exposures may favour conception of one sex over the other. During pregnancy, one sex may be more vulnerable to toxicants, thus leading to an increased risk of spontaneous abortion. Thus, although PCBs and DDE are persistent, we might not have measured the concentrations at the optimal time point, which could lead to increased imprecision.

In the present study, 11% of the samples in Cohort 1 and 0.4% of the samples in Cohorts 3 and 5 had concentrations of DDE, or at least one PCB, below the LOD. For these samples, we used a value of LOD/2, which is a simplified method. However, when less than 50% of the samples are below the LOD, this method produces minimal bias [46]. Another limitation of the present study is that the different exposures correlate moderately to strongly, meaning that we cannot separate the effects completely. Thus, we cannot determine with certainty if the associations can be ascribed to one or all of the exposures.

A strength of the present study is that our results were based on prospective birth cohorts in the Faroe Islands, where high participation rates and minimal problems with selection bias or confounding from socioeconomic factors have been documented [17]. To our knowledge, our study is the largest one so far assessing the association of directly measured mercury, ΣPCB and DDE with the secondary sex ratio. Furthermore, most of the previous studies that measured PCBs and DDE to examine associations with the sex ratio did not adjust for lipids. Serum concentrations of PCB and DDE could vary considerably based on lipid contents in the blood and not adjusting for lipids could, therefore, have increased the imprecision of the results when measuring the exposures in serum. In Cohort 1, the lack of individual lipid concentrations could affect the precision of the pollutant concentrations, but lipids in cord blood are usually low and fairly stable [15]. The imprecision is, thus, minimal and we additionally performed a sensitivity analysis adjusting for maternal BMI, which did not substantially change the estimates.

In conclusion, we found higher prenatal exposures to ΣPCB, *p,p’*-DDE and methylmercury to be associated with slightly increased secondary sex ratios in a population with a tradition for high consumption of marine food and high serum concentrations of PCB and DDE. However, our results did not take into account paternal exposure, which might also contribute to the outcome.
